# Supplementary material for: Dental Caries, Oral Health Behavior, and Living Conditions in 6–8-Year-Old Romanian School Children
Source: Children (Basel). 2022 Jun 16;9(6):903. doi: 10.3390/children9060903 (PMC9222191; doi:10.3390/children9060903)
Supplement: Supplementary file 1 [file children-09-00903-s001.zip › children-1732606-supplementary.pdf]

Fresh fruit	***Gender***	***Father's education***	***Mother's education***	***Residence***	***Type of city***
Pearson Correlation	.068	.064	.036	-.036	.012
Sig. (2-tailed)	.058	.074	.309	.307	.741
N	779	777	781	786	786

Table S1. *Correlation between fresh fruit consumption and children's demographics*

Biscuits, cookies, creme, sweet pies, sweet buns	***Gender***	***Father's education***	***Mother's education***	***Residence***	***Type of city***
Pearson Correlation	-.029	***-.179****	***-.191****	***-.096****	-.064
Sig. (2-tailed)	.415	***.000***	***.000***	***.007***	.073
N	775	773	777	782	782

Table S2. *Correlation between the consumption of biscuits, cakes, cream, sweet pies, buns and children's demographics*

Sweetened drinks	***Gender***	***Father's education***	***Mother's education***	***Residence***	***Type of city***
Pearson Correlation	-.026	***-.321****	***-.414****	***-.244****	**-.156****
Sig. (2-tailed)	.476	***.000***	***.000***	***.000***	**.000**
N	775	773	777	782	782

Table S3. *Correlation between the consumption of sweetened soft drinks and the demographic data of children*

Chewing gum with sugar	***Gender***	***Father's education***	***Mother's education***	***Residence***	***Type of city***
Pearson Correlation	-.030	***-.312*****	***-.402*****	***-.269*****	**-.223****
Sig. (2-tailed)	.403	***.000***	***.000***	***.000***	**.000**
N	765	763	767	772	772

Table S4. *Correlation between sugar consumption of chewing gum and children's demographics*

Sweets/ candies	***Gender***	***Father's education***	***Mother's education***	***Residence***	***Type of city***
Pearson Correlation	.019	*-.206***	***-.223*****	***-.103*****	**-.106****
Sig. (2-tailed)	.605	*.000*	***.000***	***.004***	**.003**
N	774	772	776	781	781

Table S5. *Correlation between the consumption of sweets / candies and the demographic data of children*

Sweetented milk with sugar/honey	***Gender***	***Father's education***	***Mother's education***	***Residence***	***Type of city***
Pearson Correlation	.039	***-.216*****	***-.240*****	***-.141*****	**-.100****
Sig. (2-tailed)	.280	***.000***	***.000***	***.000***	**.005**
N	768	767	770	774	774

Table S6. *Correlation between milk / sugar / honey consumption and child demographics*
